# Letter to the Editor, regarding the article "German Cardiac arrest Registry: rationale and design of G-CAR"

**DOI:** 10.1007/s00392-022-02130-y

**Published:** 2023-01-24

**Authors:** Jan-Thorsten Gräsner, Jan Wnent, Andreas Bohn, Berthold Bein, Stephan Seewald, Patrick Ristau, Sigrid Brenner, Matthias Fischer

**Affiliations:** 1 Institute for Emergency Medicine, University Hospital Schleswig-Holstein, Kiel, Germany; 2 Department for Anaesthesiology, University Hospital Schleswig-Holstein, Kiel, Germany; 3 German Resuscitation Registry, C/O DGAI, Nuremberg, Germany; 4 School of Medicine, University of Namibia, Windhoek, Namibia; 5 Fire Department, City of Münster, Münster, Germany; 6 Department of Anesthesiology, Intensive Care and Pain Medicine, University Hospital Münster, Münster, Germany; 7 Department for Anaesthesiology, Asklepios Hospital Hamburg St. Georg, Hamburg, Germany; 8 Department for Anaesthesiology, University Hospital Dresden, Dresden, Germany; 9 Department for Anesthesiology, ALB-FILS Hosptial, Göppingen, Germany

Sirs:

We agree with Pöss et al. [1] that proper documentation of patients suffering from cardiac arrest is key to improving outcomes. Therefore, the German Resuscitation Registry (GRR) was established some 15 years ago. Unfortunately, the authors fail to adequately describe the scope, goals, and scientific achievements of the widely implemented and established GRR.

First: The “German Resuscitation Registry” exists since 2007 and is the largest registry in Germany and one of the largest registries in Europe covering the treatment of patients with out-of-hospital cardiac arrest (OHCA). It includes 164,750 patients with treatment of out-of-hospital cardiac arrest as of August 9, 2022. As a prospective registry, it includes preclinical, clinical, and post-clinical variables as well as treatment by medical emergency teams after in-hospital cardiac arrest. In total, the GRR includes more than 400,000 datasets from OHCA, IHCA, and in-hospital emergency treatment, respectively. Using the search term "german resuscitation registry" in the title and abstract search, more than 35 publications can be found in PUBMED and more than 200 on the webpage www.reanimationsregister.de to date.

Second: Since 2016, annual reports on out-of-hospital cardiac arrests in Germany including resuscitation incidence and discharge rate with good neurological recovery according to the resuscitation guidelines have been published and made publicly available. In particular, we refer to the reports of the German Cardiac Arrest Centres, which are available since 2019, and report on diagnostics and therapy performed within the hospital as well as on long-term patients’ outcome.

Third, Pöss et al. claim that G-CAR is the first national registry including a long-term follow-up for OHCA patients. However, the German Resuscitation Registry provides discharge and 1-year survival rates as well as neurological recovery using CPC and mRS status already since 2007. In addition, quality of life after the event can be documented in the form of the EQ-5D or the SF-12 [2–5].

## Data Availability

Data sharing is not applicable to this article as no datasets were generated or analyzed for this letter to the editor.
